# Characterisation of 20th Century Cementitious Materials from Selected Cultural Heritage Structures in Slovenia

**DOI:** 10.3390/ma16186206

**Published:** 2023-09-14

**Authors:** Mateja Golež, Vesna Zalar Serjun, Mateja Štefančič, Darja Rant, Sabina Dolenec

**Affiliations:** Slovenian National Building and Civil Engineering Institute, 1000 Ljubljana, Slovenia; vesna.zalar@zag.si (V.Z.S.); mateja.stefancic@zag.si (M.Š.); darja.rant@zag.si (D.R.); sabina.dolenec@zag.si (S.D.)

**Keywords:** 20th century, cementitious materials, historical concrete, cultural heritage

## Abstract

This paper deals with the characterisation of cementitious materials from selected cultural heritage structures in Slovenia. The mineralogical–petrographic compositions of an aggregate, a type of binder and secondary minerals were studied via electron microscopy and X-ray powder diffraction. The porosity and pore network were determined using a mercury porosimeter. The results show that the aggregate of the samples was highly diverse, ranging from limestone, dolomite, quartz, feldspar and mica. The binder of the investigated samples was cementitious; either ordinary Portland cement was used, or ordinary Portland cement blended with ground granulated blast furnace slag was used. Some samples consisted of cement–lime binders. The investigated examples entailing cement materials for their construction contribute to a better understanding of the technology used to prepare historical cementitious and cement–lime mixtures.

## 1. Introduction

The invention and patenting of Portland cement have opened wide possibilities for the construction of high and lean buildings, dome-shaped elements of large dimensions, bridges, water barriers, water reservoirs, curtain walls and roof covering and the production of urban furniture, concrete plastic and diverse decorative elements [1]. Hence, modern Portland cement (raw meal burnt with a temperature of up to 1450 °C) slowly replaced natural cement (burnt from marlstone with a low CaO content; burnt with a temperature in the range from 800 °C to 900 °C) in the 19th century [2,3,4].

Cementitious materials were already noted as contemporary materials in the Roman Empire [5], but were first widely noted through the works of the French architect of the 20th century modern movement La Corbusie [6]. His Slovenian contemporaries were the architects Maks Fabiani (1865–1962) and Jože Plečnik (1872–1957), who created a rich opus of modern 20th century architectural heritage in Slovenia, Vienna (Austria) and Prague (Czech Republic) with their innovative style in architecture. Another important architect for the 20th century architectural heritage was Edvard Ravnikar (1907–1993), who prominently influenced Slovenian architecture after World War II. Slovenian experts, therefore, strive to preserve their amazing works in line with the international activities conducted within the scope of the DOCOMOMO (International Committee for Documentation and Conservation of Buildings, Sites and Neighbourhoods of the Modern Movement) and pursuant to the national regulations governing the protection of 20th century architectural heritage [7]. The architectural monuments of the architect Jože Plečnik are included in the UNESCO World Heritage List from 2021 [8]. To better research and protect 20th century architectural heritage in Slovenia, the Slovenian National Building and Civil Engineering Institute established the 20th Century Architectural Heritage Research Laboratory. The laboratory was created with the financial support of the INTERREG SI-ITA program as a result of the MACC (Modern Art Conservation Centre) project. The purpose of establishing a laboratory is to conduct interdisciplinary research on 20th century heritage buildings and historical concrete, including the recycling of historical concrete like tiles from Kuretno near Laško [9]. This makes Slovenia one of the first countries in the world to professionally protect the architectural heritage of the 20th century and to take into account a circular economy for the sustainable renovation of cultural heritage buildings, which are modern trends in research [10,11,12]. The systematic research of recycled cementitious materials at the macro and micro levels is carried out as the recycled aggregate must meet the requirements of the standard for construction products [13,14]. Furthermore, the presence of asbestos is checked, which was added to cementitious materials in the past to improve the mechanical characteristics, but today, this practice is no longer followed because it was proven to have a harmful effect on people’s health [15].

The construction of buildings using cementitious materials stems from the mineral resources that are used for burning cement clinker and the early development of the cement industry, which dates back to 1876, when the first cement factory in Trbovlje, Slovenia was built [16]. Cementitious binder and the related preparation of various cementitious mixtures paved the way for the use of cementitious materials in Slovenian construction relatively early compared to England, where Portland cement was patented in 1824 [17,18]. Cementitious materials were frequently used as building materials for construction elements or ornaments (interior and exterior), whereby wooden panelling and various colours and aggregate grading were used to emphasise the aesthetic strength of concrete as visible concrete [19]. The diverse compositions of cementitious material mixtures are also reflected at the micro level and result from the development of various cement clinker burning technologies developed over time [20,21,22].

To evaluate the conditions of the cementitious materials, samples were taken for laboratory study to determine the materials’ components and compositions. This made it difficult to replicate their composition in terms of the source and nature of the input materials like the aggregate type, the aggregate size and the shape and type of binder.

This article includes results from a preliminary study that aims to inspect the microstructures of selected samples belonging to unique modern architectural heritage structures of various architectural types, periods and geographic areas to create the first database on the compositions of cementitious materials for further conservation interventions of heritage buildings and cementitious products, like cement-based roof tiles.

## 2. Materials and Methods

Cementitious material samples were taken from the exteriors and interiors of the buildings, which were built between 1881 and 1978 (Table 1). Seven samples of historical cementitious materials were studied. The examined samples of historical cementitious materials were marked with a serial number, as they were taken as a part of all samples from the MACC project, where we focused on all materials of the architectural heritage of the 20th century. One sample was taken from a roofing tile from the Church of St. Michael in Črna vas at Ljubljana, built by architect Jože Plečnik (M6); one sample was from a commercial building hosting a store and post office in the village of Vremski britof in the region of Kras, built by architect Edvard Ravnikar (M17); two samples were from structures of works of architect Maks Fabiani—one from the abandoned Miramonti Hotel in Štanjel (M19) in the region of Kras and the other from the water distribution system of the Ferrari Garden in Štanjel (M20); and three samples were taken from structures of unknown architects—the Arrigoni factory in Izola on the Slovenian coast (M4), the water barrier (dam) on the Sedučnik stream in NW Slovenia (M5) and a sample from a concrete roofing tile, which used to cover a vernacular architecture building in the village of Kuretno near Laško in Central Slovenia (M9).

The selected samples were taken in a destructive manner and in accordance with the conservation–restoration method. This prescribes a minimum sample collection, which, on the one hand, must not cause significant damage to the building, and on the other hand, must have credible and useful research results for further conservation–restoration interventions. The samples of cementitious materials that were included in the study were therefore taken from buildings that are partially damaged and are scheduled for conservation–restoration intervention. It was only possible to take a larger number of samples for the sample of the concrete roof tile, M9, as all the roof tiles were removed from the building and replaced with new ones. Although modern standards for ensuring the representativeness of cementitious materials are prescribed, we cannot always follow them in the conservation–restoration practice because we are limited by the number of samples that can be taken from the monument [23].

**Figure 1:**
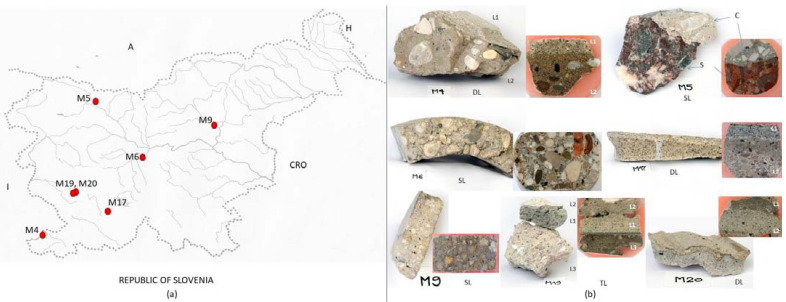
(**a**) Locations of historical structures where samples of cementitious material were taken. (**b**) Investigated cementitious material samples with fresh fracture lines and polished ground joints (M—sample, L—layer, SL—single layer, DL—double layer, TL—triple layer, C—concrete, S—stone).

Information on the investigated samples is given in Table 1, and the samples are shown in Figure 1.

The polished cross sections of the samples were examined using a scanning electron microscope (SEM), JEOL 5500 LV SEM, equipped with Energy Dispersive X-ray Spectrometry (EDS) in a low vacuum mode (between 10 and 15 Pa) at an accelerating voltage of 20 kV and a working distance of 20 mm. The correction of EDS data was performed based on the standard ZAF correction procedure included in the INCA Energy 200 software (Inca, Oxford Instruments, Oxford, UK). 

The X-ray powder diffraction analysis (XRD) of the samples was performed by using an Empyrean (Panalytical, Malvern, UK) diffractometer. The radiation source used was a copper anode. The recording was conducted under a voltage of 45 kV, a current of 40 mA, a speed of 1°/min and in the range of 5–70° 2θ. The interpretation of results was performed using the HighScore v.4.x LTU software and the PDF-4 database. Prior to the XRD analysis, the samples were pulverised in an agate grinding wheel to a grain size of <40 μm. The samples were mounted into the 27 mm diameter sample holders using the backfilling technique.

The investigation of the microstructures of historical concrete samples was performed on a field emission scanning electron microscope (FE-SEM, ULTRA plus, Zeiss, Jena, Germany) operating at an accelerating voltage of 13–17 kV, an aperture diameter of 30 µm, a working distance of 5–7 mm, a magnification of 5–50 kX, a specimen chamber pressure of 2 × 10^−5^ Pa and with Inlens or SE2 detectors. A crushed sample was mounted on a sample holder with carbon tape and additionally sputter-coated with gold SCS 005 Sputter Coater (Bal-Tec AG, Balzers, Liechtenstein) to prevent the charging of the sample surface. The chemical composition of selected areas was investigated with an EDS detector and acquisition program Inca (Oxford Instruments, Oxford, UK).

The pore systems of the samples were further investigated by means of mercury intrusion porosimetry (MIP). Two fragments, approximately 2 cm^3^ in size, were analysed using Micromeritics^®^ Autopore IV 9500 equipment (Norcross, GA, USA). Prior to analyses, samples were stored for seven days in approx. twenty times their volume of isopropanol. For the first two days, isopropanol was replaced two times per day, and from then on, it was replaced once per day. After that, isopropanol was removed via oven drying at 105 °C [24]. The samples contained aggregate as well as binder and were analysed within the range of 0 to 414 MPa using penetrometers for solid samples.

## 3. Results

### 3.1. SEM/EDS Analysis

Stratigraphically, we distinguish between single-layer (M5, M6 and M9), double-layer (M4, M17 and M20) and triple-layer (M19) applications of historical cementitious materials (Figure 1). The investigated samples are heterogeneous mixtures of carbonate aggregate (limestone and dolomite), quartz, grains of mica, feldspars and cementitious or cement–lime binder with frequent clinker remnants and ground granulated blast furnace slag. 

#### 3.1.1. Aggregate

**Figure 2:**
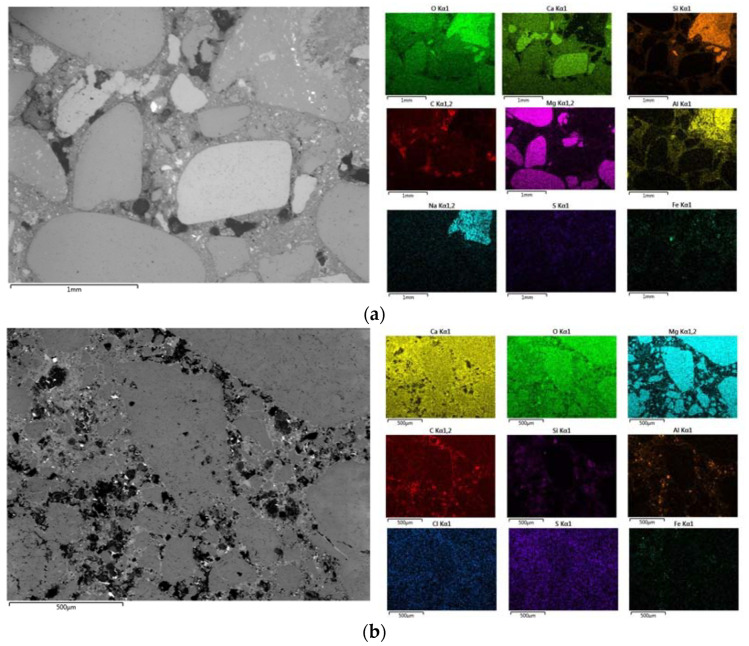
(**a**) Mapping analysis of sample M6 with heterogeneous composition of aggregate. (**b**) Mapping analysis of sample M9 with homogeneous composition of aggregate.

Single layer cementitious materials are cementitious materials that are applied in an external environment. The investigated sample M5 is a mass concrete water dam sample, while samples M6 and M9 are concrete roof tile samples. The aggregate in sample M5 is a mixture of dolomite grains, limestone and quartz. The grains are angular to rounded, and the largest grain in the examined sample is 12 mm. The aggregate grains of sample M6 are also a mixture of aggregate grains of limestone, dolomite and quartz, and lithic grains. The grains are round in shape, with the largest aggregate grain being 13 mm. The aggregates in sample M9 are composed of dolomite, angular in shape and have a maximum grain size of 4 mm. Figure 2a,b shows the differences in the compositions and shapes of the aggregate grains of the two samples of roof tiles, M6 and M9, respectively.

The double layer finishing layers represented by samples M4 (M4/L1 and M4/L2), M17 (M17/L1/M17/L2) and M20 (M20/L1 and M20L2) are characterised by the fact that the bottom layer is made of the coarse aggregate fraction and the upper layer is made from fine aggregate. In sample M4, layers L2 and L1 are aggregate grains composed of limestone and dolomite, and individual feldspar grains are also present in layer L1. The grains are angular to rounded, and they differ in their maximum grain size, which is 10 mm in the L2 layer and 2 mm in the L1 layer. In the cement material sample M17, the aggregate grains in layers L1 and L2 are composed of quartz and limestone. In terms of shape, the aggregate grains are square-edged to rounded, but they differ in the maximum grain size, which is 4 mm in the L2 layer and 1 mm in the L1 layer. Even layers L1 and L2 in sample M20 do not differ in the composition of aggregate grains because they consist of limestone, dolomite and quartz grains. In terms of shape, the grains are angular to rounded and very fine, since in the L2 layer, the maximum grain size is 1 mm, while in the L1 layer, the maximum grain size is 0.1 mm. The three layers of the M19 sample have a very similar composition. The composition of the aggregate grains in layer L3 is composed of quartz, limestone, dolomite and mica, and the composition of the aggregate grains in layer L2 is composed of limestone, dolomite and lithic grains of feldspar and quartz, while the composition of layer L1 is composed of limestone, feldspar, quartz and mica. The grains in all three layers are rounded to angular, and they differ in size, as the maximum grain size in layer L3 is 5 mm, the maximum grain size in layer L2 is 2 mm and the maximum grain size in layer L1 is 0.5 mm (Table 2).

#### 3.1.2. Binder

Two types of binders, OPC and OPC with the addition of lime, were identified in the investigated groups of cementitious materials. OPC with the addition of lime is present in samples M4/L1 and M19/L2, while in the other samples, only the OPC binder is present.

**Figure 3:**
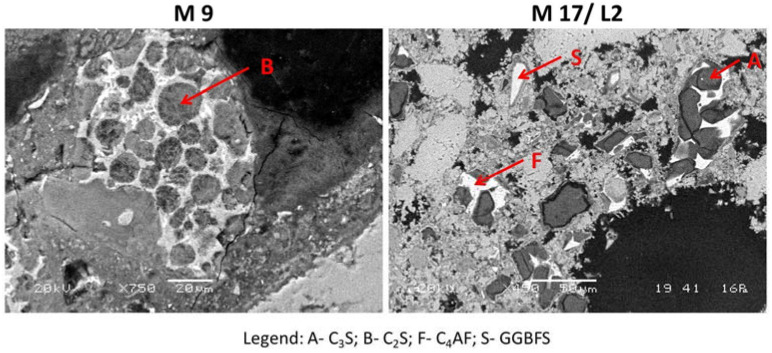
SEM/BSE micro-images of historical cementitious material samples with heterogeneous composition of clinker remnants and GGBFS.

The historical cementitious material samples M4 (L1 and L2), M17 (L1 and L2) and M19 (L1, L2 and L3) contain grains of artificial pozzolans of ground granulated blast furnace slag (GGBFS). The contact between the cementitious matrix and pozzolans is represented by a clearly expressed reaction rim, which shows the effect of GGBFS supplementary cementitious material (Figure 3). GGBFS is a by-product of the metallurgical industry. The latent hydraulic reactivity of ground granulated blast furnace slag was discovered at the end of the 19th century by Emil Langen [25]. The reactive properties of GGBFS are the result of the amorphous phase, which is achieved by the rapid cooling of the slag. Studies so far have shown that the hydraulic properties of slags depend on their production systems and chemical compositions, and the reaction mechanisms between GGBFS and Portland cement are also complex [26,27]. The grains of unhydrated cement clinker remnants are present in all the investigated samples. Their sizes range between 50 µm and 250 µm. Under a high magnification under an electronic microscope, one may see the skeletal structures of the clinker remnants with individual crystal phases of tricalcium silicate–alite (C_3_S), dicalcium silicate–belite (C_2_S), aluminate (C_3_A) and ferrite (C_4_AF). The microstructural heterogeneity of clinker remnants is recognised for euhedral crystalline structures of alite, with hexagonal crystalline grains and subhedral crystalline grains of round belite grains (Figure 3). As interstitial phases, the aluminate and ferrite phases occur, which differ from one another by the intensity of bright colour, ranging from grey to white, depending on the weight of the atoms. The heavier the atoms, the brighter the grey in the observed phase (Figure 3). The surfaces of the ferrite phase are brighter than alite under an electronic microscope, and the aluminate is brighter than belite [28].

### 3.2. X-ray Powder Diffraction

The identified mineral phases of the studied samples defined via XRD may be sorted into three broader groups according to their origin. The primary mineral phases represent constituents of aggregate, while the secondary mineral phases may be divided into the mineral phases representing hydration products and the mineral phases formed during the degradation processes (Table 3). The first group involves calcite, dolomite, quartz, feldspars, illite/muscovite and clinochlore as constituents of the aggregate fraction. The second group includes hydration products, such as portlandite, ettringite and hydrocalumite. The third group includes secondary minerals, such as gypsum, vaterite and calcite. The XRD patterns of the samples analysed also show that the mineral calcite is present in all the historical cementitious material samples, vaterite is present in the M4/L2, M5, M6 and M19/L1 samples, while aragonite is present in the M19/L3 sample (Table 3). 

**Figure 4:**
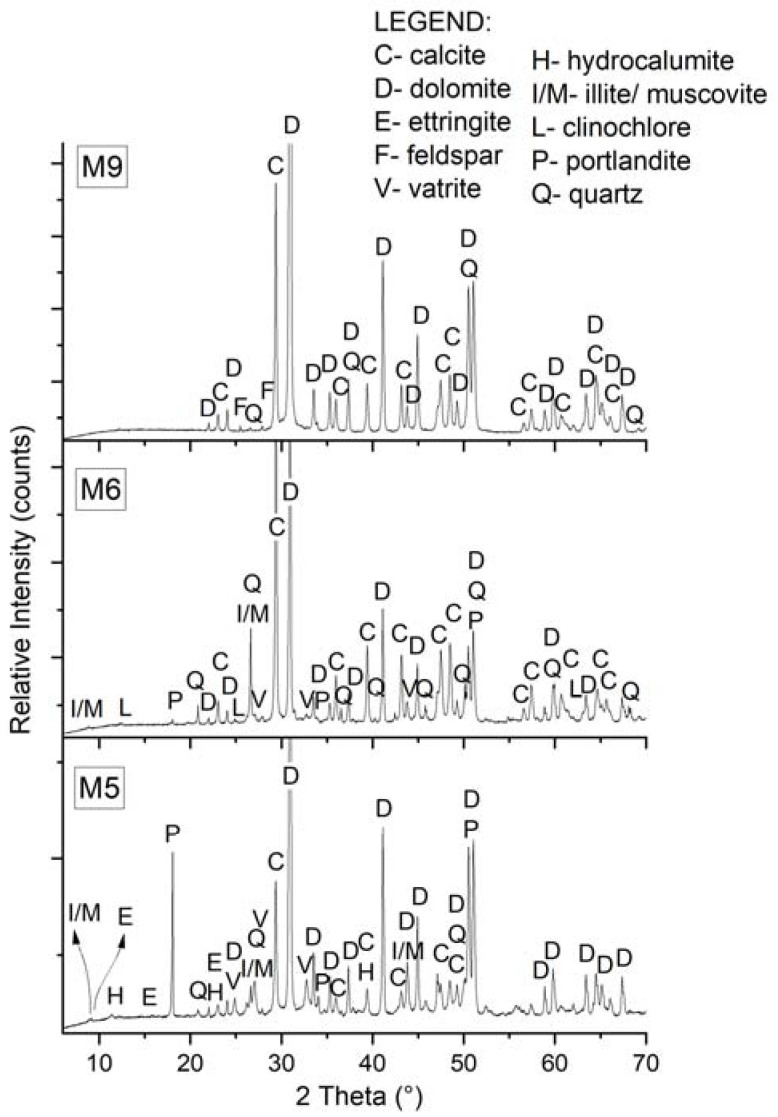
XRD patterns of single-layer samples M5, M6 and M9.

The presence of calcite in the investigated samples of historical concrete is attributed to the aggregate, the carbonation of portlandite and the C–S–H gel and to the recrystallisation processes [29,30]. The carbonation of the C–S–H gel may also be the cause for the crystallisation of vaterite and aragonite [30]. Carbonation (self-healing) is a process where the atmospheric carbon dioxide reacts with the hydrated cement in the presence of moisture, while the crystallisation products affect the porosity of the material, the transition of fluids and gases in the cement paste, the micro and macro mechanical changes and the shrinking and cracking of concrete [30]. As an example of XRD analysis, we provide a mineralogical image of the samples of the single-layer cement materials M5, M6 and M9, which were exposed to external environmental factors and therefore to intensive carbonisation processes (Figure 4).

### 3.3. FE/SEM

**Figure 5:**
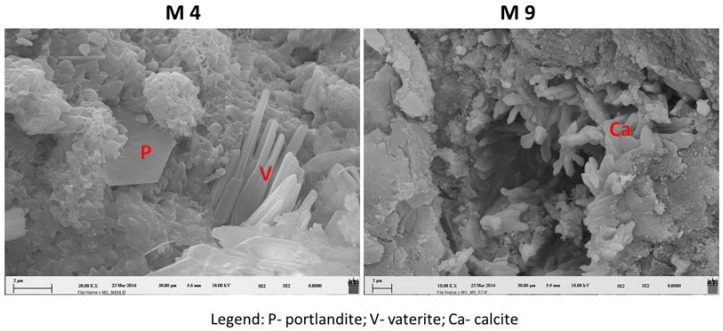
Field emission scanning electron microscopy of carbonation and hydration processes (M4) and recrystallisation of crystals (M9) of historical cementitious materials.

The self-healing process was also checked on the selected samples of cement materials using the FE/SEM method. Investigations were carried out on fresh fractures, where the spatial pattern of the crystallisation of the secondary minerals was revealed, as can be seen in Figure 4. Using this method, we confirmed the findings of the presence of secondary minerals, which were identified via XRD analysis. Figure 5 shows a spatial image of the secondary minerals resulting from carbonation, and vaterite as well as calcite resulting from the recrystallisation of the cement matrix. Portlandite crystals are also present in the cement matrix as a result of hydration processes.

The penetration of CO_2_ in a cementitious material depends on the quality of cementitious material compaction and on the microporosity of the hydrated binder, which influences the carbonisation process.

### 3.4. Porosity and Pore Size Distribution

Table 4 shows the parameters determined via mercury intrusion porosimetry (total porosity, average pore diameter, bulk density and apparent density), whereas Figure 5 shows the representative pore size distribution.

The porosity values ranged from 13.1 to 32.0%. The bulk densities of the samples ranged from 1.73 g/mL to 2.30 g/mL, whereas the apparent densities were within the range of 2.48 g/mL to 2.66 g/mL (Table 4).

**Figure 6:**
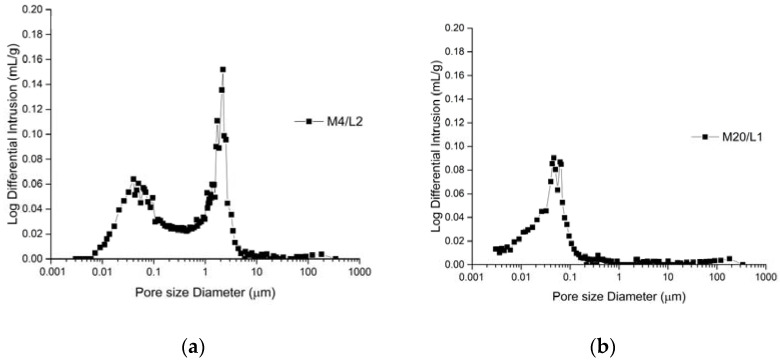
(**a**) Pore size distribution for the cement-lime mortar M4/L2; (**b**) cement render (M20/L1).

With the exceptions of samples M19/L1–L3 and M20 L1 that show a unimodal distribution of pores, a bimodal distribution of pores was characteristic for the other samples. The main intrusion peak was observed in the range from 0.7 to 1.6 µm for sample M19 and was shifted to smaller pores for sample M20/L1 (Figure 6). Regarding the samples with a bimodal distribution of pores, for the cementitious binder, the fist peak was observed in the range of 0.01 and 45.3 µm, and the second peak was observed in the range from 0.07 to 60.4 µm. For instance, sample M4 shows a minor intrusion peak at around 0.05 μm and a larger intrusion peak at around 2.2 μm for the coarse-grained layer (M4/L2), which is, however, shifted towards larger pores in the fine-grained layer (M4/L1), with peak of 0.5. While the coarse-grained layer consisted of a cementitious binder, the fine-grained layers also contained lime. 

Sorption pores (<0.1 μm) are gel pores that develop in hydrated phases, and they are related to the presence of hydraulic phases as C–S–H (M5 and M20/L1 had the most pores below 0.1 μm). However, for the M4/L1, M17 and M19 samples, pores smaller than 0.1 μm were in limited amounts. 

Capillary pores, the diameter of which range from 0.1 to 100 µm, are formed in the binder matrix and in the transition zones during the hydration and setting process or due to the removal of water during hardening. The water/binder ratio and the carbonation/hydration rate influence the percent of capillary pores [31].

Smaller capillary pores (between 0.1 and 1 µm) were observed for samples M5, M20 (in addition to gel ones) and M19. Samples M4/L1, M17/L1 and L2 contained capillary pores between 0.15 and 60.4 µm.

Large capillary pores, sometimes considered as those over 50 μm and sometimes considered as those over 4–50 μm are formed in the spaces between the binder and the aggregate.

## 4. Discussion

This article includes results from a preliminary study that aims to inspect the microstructures of selected specimens from Slovenia belonging to unique modern architectural heritage structures of various architectural types, periods, and geographic areas. In this way, the first step to creating a database on the composition of cementitious materials for further conservation interventions of heritage buildings and cementitious products would be achieved.

The investigated samples of historical cementitious materials are heterogeneous mixtures prepared with different aggregates and binders, and in some cases, are also prepared with some pozzolanic additives like granulated ground blast furnace slag. According to the examined samples of cementitious materials, there is a trend of adding ground blast furnace slag in the buildings in the SW part of Slovenia, but there will be a need for a further systematic study of these materials so that we can also define the trend geographically according to the origin of the cement clinker.

The historical cementitious materials demonstrate pronounced heterogeneity, including in the size and amount of clinker remnants prevailing in the cementitious materials prepared with a fine-grained aggregate. Furthermore, the level of carbonation is highly pronounced, which is reflected in the carbonation products, ranging from vaterite and aragonite to calcite.

Differences were also noted in porosity with respect to the type of binder used. Samples in which a lime binder was added to a cementitious binder have the highest porosity values.

For the purposes of conservation and restoration protocols for the maintenance of contemporary architectural heritage assets, the prepared cementitious materials should be similar to the identified composition, unless marine aggregate was used. For instance, since cementitious roof tiles do not contain components that would adversely affect their characteristics or do not affect human health like asbestos does, they can be recycled. Following the principles of the sustainable renovation of cultural heritage buildings, the aggregate from the original roof tiles may be used in the new roof tiles. In this way, we contribute to reducing the use of natural resources and at the same time preserve the original materials.

## Figures and Tables

**Table 1:** Investigated samples of historical cementitious materials with their locations and period of construction.

Sample	Structure/Architect	Sampling Location	Period
M4	Arrigoni factory (industrial heritage), Izola/unknown	Reinforced concrete lintel (interior concrete, mortar)	1881 and 1936–1938
M5	Dam, Sedučnik stream, Dovje/unknown	Stream bed (exterior concrete)	early 20th century
M6	St. Michael’s Church, Črna vas/ Jože Plečnik	Roof (concrete tile) (exterior)	1937–1939
M9	Vernacular architecture, Kuretno near Laško/unknown	Landfill (waste concrete tile)	early 20th century
M17	Post office and shop, Vremski Britof/ Edvard Ravnikar	South wall, entrance (exterior render)	1978
M19	Miramonti Hotel, Štanjel/ Maks Fabiani	Second floor (interior masonry plaster)	early 20th century
M20	Ferrari Garden, Štanjel/ Maks Fabiani	Water tank, entrance (facade render)	1923–1935

**Table 2:** Results of the analysis of aggregate and binder type in historical cementitious materials.

Sample	Max Aggregate Grain Size (mm)	Aggregate Type	Aggregate Shape	Binder
M4/L1	2	limestone, dolomite and feldspar	angular and round	OPC and slag lime
M4/L2	10	limestone and dolomite	round and angular	OPC and slag
M5	12	dolomite, limestone and quartz	angular and round	OPC
M6	13	limestone, dolomite, quartz and lithic grains	round	OPC
M9	4	dolomite	angular	OPC
M17/L1	1	quartz and limestone	angular and round	OPC and slag
M17/L2	4	limestone and quartz	angular and round	OPC and slag
M19/L1	0.5	limestone, feldspar, quartz and mica	round and angular	OPC, lime and slag
M19/L2	2	limestone, dolomite, feldspar, lithic grains and quartz	angular and round	OPC and slag
M19/L3	5	quartz, limestone, dolomite and mica	angular and round	OPC and slag
M20/L1	filer	limestone, dolomite and quartz	angular and round	OPC
M20/L2	1	limestone, dolomite and quartz	angular and round	OPC

**Table 3:** Results of X-ray powder diffraction analysis.

Mineral/Sample	M4/1	M4/2	M5	M6	M9	M17/2	M19/1	M19/3	M20/1	M20/2
calcite	✓	✓	✓	✓	✓	✓	✓	✓	✓	✓
dolomite	✓	✓	✓	✓	✓	/	✓	✓	✓	✓
feldspar	✓	✓	/	/	✓	/	✓	/	✓	✓
quartz	✓	✓	✓	✓	✓	✓	✓	✓	✓	✓
illite/muscovite	✓	/	✓	✓	/	/	/	/	✓	✓
clinochlore	✓	✓	/	✓	/	/	/	/	/	
vaterite	/	✓	✓	✓	/	/	✓	/	/	/
aragonite	/	/	/	/	/	/	/	✓	/	/
gypsum	/	✓	/	/	/	/	✓	✓	/	/
portlandite	/	/	✓	✓	/	/	/	/	/	
ettringite	/	/	✓	/	/	/	/	/	/	
hydrocalumite	/	/	✓	/	/	/	/	/	/	✓

**Table 4:** Porosity, bulk density, apparent density and average pore diameter of historical cementitious materials.

Sample	Porosity (%)	Bulk Density (g/mL)	Apparent Density (g/mL)	Average Pore Diameter (µm)
M4/L1	32.0	1.73	2.54	0.35
M4/L2	22.7	2.02	2.61	0.08
M5	13.6	2.30	2.66	0.04
M6	13.2	2.21	2.55	0.05
M9	14.2	2.28	2.65	0.06
M17/L1	16.3	2.08	2.48	0.05
M17/L2	17.2	2.12	2.56	0.09
M19/L1	27.9	1.88	2.61	0.08
M19/L2	20.2	2.08	2.61	0.14
M19/L3	29.3	1.82	2.57	0.21
M20/L1	13.1	1.96	2.25	0.2
M20/L2	22.2	1.90	2.44	0.2

## Data Availability

Not applicable.
